# Correction: Wireless recording from unrestrained monkeys reveals motor goal encoding beyond immediate reach in frontoparietal cortex

**DOI:** 10.7554/eLife.69225

**Published:** 2021-04-13

**Authors:** Michael Berger, Naubahar Shahryar Agha, Alexander Gail

Berger M, Agha NS, Gail A. 2020. Wireless recording from unrestrained monkeys reveals motor goal encoding beyond immediate reach in frontoparietal cortex. *eLife* **9**:e51322. doi: 10.7554/eLife.51322.

Published 4, May 2020

We inadvertently used the wrong colormap for Figure 3A so that the colors were inconsistent with the text, legend, and other figures. We corrected the figure. Text and legends remain unchanged as they already described the intended colors.

The corrected Figure 3 is shown here:

**Figure 1:**
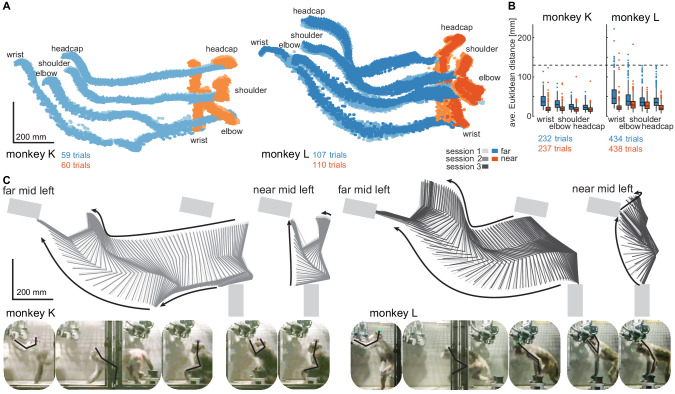


The originally published Figure 3 is shown for reference:

**Figure 2:**
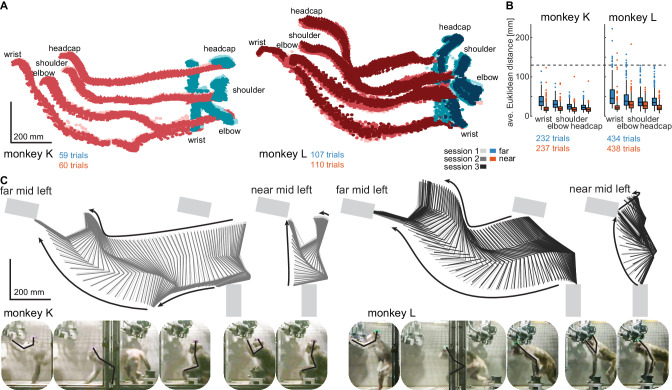


The article has been corrected accordingly.

